# How useful is the concept of the ‘harm threshold’ in reproductive ethics and law?

**DOI:** 10.1007/s11017-014-9302-8

**Published:** 2014-08-09

**Authors:** Anna Smajdor

**Affiliations:** Norwich School of Medicine, University of East Anglia, Norwich, NR4 7TJ UK

**Keywords:** Reproductive technology, IVF, Parfit, Harm, Non-identity, Kant

## Abstract

In his book *Reasons and Persons*, Derek Parfit suggests that people are not harmed by being conceived with a disease or disability if they could not have existed without suffering that particular condition. He nevertheless contends that entities *can* be harmed if the suffering they experience is sufficiently severe. By implication, there is a threshold which divides harmful from non-harmful conceptions. The assumption that such a threshold exists has come to play a part in UK policy making. I argue that Parfit’s distinction between harmful and non-harmful conceptions is untenable. Drawing on Kant’s refutation of the ontological argument for God’s existence, I suggest that the act of creation cannot be identical with the act of harming—nor indeed of benefiting—however great the offspring’s suffering may be. I suggest that Parfit is right that bringing children into existence does not usually harm them, but I argue that this must be applied to *all* conceptions, since Parfit cannot show how the harm threshold can be operationalised. If we think certain conceptions are unethical or should be illegal, this must be on other grounds than that the child is harmed by them. I show that a Millian approach in this context fails to exemplify the empirical and epistemological advantages which are commonly associated with it, and that harm-based legislation would need to be based on broader harm considerations than those relating to the child who is conceived.

## Introduction

Many people believe that the ethics of new reproductive technologies can be ascertained by establishing whether they would be harmful to offspring. It is similarly often assumed that the legal status of such technologies follows straightforwardly from this: those that are harmful are unethical and should be illegal. Reproductive cloning, for example, might result in children who suffer from genetic anomalies, and therefore, it is widely deemed unethical. Similarly, the deliberate creation of embryos with disabilities is commonly regarded as harmful and unethical. Accordingly, both practices are illegal in UK legislation and many other jurisdictions [1–3].

It might seem unnecessary to unpick the moral basis on which these reproductive possibilities should be deemed unethical or illegal—especially since it is so widely agreed that they should obviously be so. However, my aim in this paper is to show that some of the most widely accepted assumptions are in fact false and that the apparent epistemological advantages of harm-based legislation are illusory in this context. The failure to specify more accurately and convincingly what—if anything—is morally wrong about techniques such as cloning or deliberate creation of disabled offspring in fact leaves us without a coherent basis on which to legislate against such procedures. I also wish to show that some of the most aggressively rationalist approaches to these questions are fundamentally flawed.

John Harris and many others take a broadly Millian view: the law ought not to forbid individuals to pursue their interests and activities, unless those interests or activities harm others [4]. I do not share the conviction that harm to offspring is the sole focus of moral concern in reproductive decisions. However, I do have some sympathy with a Millian approach to legislation. As Arthur Ripstein argues, the harm principle and its relationship with law can be interpreted in various ways: ‘[f]or some of its defenders, the harm principle operates as a constraint on the legal prohibition of moral wrongs that have been identified independently of it. For others, such as Joel Feinberg, harm is neither a necessary nor a sufficient condition for criminalization, but “always a good reason” in favor of it’ [5]. Not all moral wrongs can feasibly be prohibited by law, but if those that involve harm to others are more identifiable and less subjective, then the harm principle may be a useful interface between morality and legislation. In short, the harm principle may function as a lowest common denominator.

However, I will argue that a Millian approach is peculiarly problematic in the context of reproductive technology. Because of this, those who regard the harm principle as the only basis on which to prohibit certain reproductive technologies will find that some of the most intuitively objectionable uses of reproductive technology, such as reproductive cloning or the deliberate creation of severely disabled offspring,^1^ fail to meet the criteria required to justify a legal ban.

## Background: the move towards harm-based legislation in the UK

The UK was the first country to develop a legal and regulatory framework for reproductive technologies. Its initial approach to legislation was based on a moral analysis of the issues involved. The Warnock Committee, which undertook this analysis, adopted an explicitly non-utilitarian position, stating that simply weighing up harms and benefits was unsatisfactory because there were deeper ethical concerns at stake [6, p. 4]. The introduction to the Warnock report quotes Hume: ‘morality is “more properly felt than judg’d of”’ [7, p. x]. Similarly Humean tones can be found in various places in the report: ‘reason and sentiment are not opposed to each other in this field’ [6, p. 1]. And ‘moral questions … involve not only a calculation of consequences, but also strong sentiments’ [6, p. 2].

The Warnock Report was widely praised for its pragmatism in developing a sound ethical framework for embryo research and fertility treatment. Yet, the Act which followed was dogged by controversies and legal challenges (see, e.g., [8]). The social and political climates were changing, and the values that the Warnock Report had espoused and argued for were repudiated on many sides. Robert Lee and Derek Morgan write that ‘established boundaries were decomposing. The nature and stability of the family and marriage were waining [sic]; the openness and acceptability of heterosexual partnerships without marriage and homosexual relationships without moment were waxing; and established social and legally enforced gender roles and stereotypes, both outside and inside the home, were being recast in the long shadows of feminism…. The institutional security … of established ethical concerns was under fundamental assault from feminisms, the collapse of traditional theological canons and the emergence of new questions in the methodology and epistemology of ethics’ [9, p. 2].

This question of epistemology is key here. The Warnock Committee explicitly allied its arguments with emotive reasoning. The idea that emotion can or should play a part in moral deliberation—and feed into subsequent legislation—is unwelcome to those who seek a more empirical and evidence-based approach. Warnock’s rejection of a utilitarian framework meant that empirical justifications and evidence were not always required or sought in order to substantiate moral claims. Philosophers and politicians of a more utilitarian bent were quick to seize on these apparent failings, and to argue for an alternative strategy. The UK’s House of Commons Science and Technology Committee (henceforward, STC) embarked on a review of the law in this area in 2004 [10, pp. 4–5]. The STC argued that modern science had already rendered Warnock’s approach obsolete [10, p. 3]. In 2005, the STC’s own report was published [10, p. 18]. It differed from its predecessor in its explicit conviction that the risk of harm associated with specific reproductive techniques can be articulated, projected, quantified, and *proven empirically* in a way that Warnock’s arguments could not.

Repudiating the Humean perspective adopted by Warnock, the STC favoured instead a Millian approach, arguing that ‘there is an obligation to avoid harm wherever possible…. The only ethical issue is what criteria should be employed’ [10, p. 19]. Throughout the report, evidence is pitted against prejudice, and reason against emotion: ‘we recommend … more detailed guidance … based on evidence not on prejudice’ [10, p. 187], and ‘the word eugenics must not be used as an emotive term of abuse to obscure rational debate’ [10, p. 55]. The STC report was also notable for its reliance on the work of John Harris, who is cited many times in the report, in contexts that emphasise Harris’s commitment to the harm principle in reproductive ethics and legislation.

For example, with regard to the use of foetal ovarian tissue (to overcome a shortage of human eggs for use in reproductive technologies), Harris asks, ‘will this knowledge [of having been born from foetal ovarian tissue] be so terrible that it would be better that no such children had ever been or were ever born?’ [10, p. 19]. Harris is also quoted in the report observing that most arguments in favour of restricting access to reproductive technologies are unjustified as they ‘do not point to dangers or harms of sufficient seriousness or sufficient probability or proximity to justify the limitation on human freedom that they require’ [10, p. 18].

Harris’s use of the word ‘sufficient’ here suggests that there is a threshold beyond which legal restrictions are justified on the basis that offspring would be harmed. Those conceptions that would *not* breach this threshold should not be illegal. If one subscribes to this view, the next task is to establish *how much* harm a child would need to suffer in order to be born on the wrong side of this threshold. In short, one must ascertain where the threshold is. This logical next step was not taken in the STC report, however. And although Harris’s own works appeal frequently to a threshold between harmful and non-harmful conceptions, he does not attempt to show where it should be set.

This is also true of many other philosophers who make use of this concept in their reasoning. John Robertson suggests that a child’s interests cannot be protected by preventing its birth *unless* ‘the harmful conditions are such that the very existence of the child is a wrong to it’ [11, p. 75]. But he does not specify at what point this occurs. Ruth Macklin asserts that ‘evidence, not mere surmise, is required to conclude that the psychological burdens of knowing that one was cloned would be of such magnitude that they would outweigh the benefits of life itself’ [2, p. 213]. But she does not attempt to provide the evidence that she says is necessary. This may be partly because she and others believe that the burden of proof lies with those who would limit reproductive freedom, as Harris has stipulated [12, p. 79]. Yet, even if we accept this point, it would be disingenuous to suppose that we could send people out to gather evidence that cloning would or would not breach the harm threshold without first having agreed at least in principle *where that threshold should be set.* Harris and others therefore cannot escape the challenge of engaging with the empirical question of where this threshold is.

## Parfit’s argument

Harris and others who make use of the harm threshold in their responses to questions of reproductive ethics draw on Parfit’s non-identity problem. Our intuitive tendency to say that a reproductive decision which results in the conception of a diseased child *harms* the child is hard to explain if the child could not otherwise have existed. The failure to locate the harm threshold, to operationalise it, and to make use of its apparent epistemological advantages in legislation are attributable, I suggest, to difficulties inherent in Parfit’s argument itself.

Parfit asserts that the act of conceiving a child with a disease or disability does not usually harm the child [13, pp. 351–377]. A woman receiving treatment for syphilis could conceive a child now or wait until she has been cured in 6 months. If she conceives now, her child will be born with syphilis. If she waits, a *different* child will be born (free of syphilis) because a different egg and sperm will be involved, resulting in a genetically different individual. The child conceived *now* could not exist without suffering from congenital syphilis. Therefore, it cannot be harmed by being brought into existence suffering from syphilis. In Parfit’s argument so far, this seems to be a matter of logic and metaphysics: there is no causal mechanism or process by which we can understand this particular child to have been harmed. And however much we might condemn the choices made by his mother, we cannot articulate our condemnation in terms of harm to the child.

Parfit argues, however, that a child born with a worse disease than syphilis *could* have been harmed—if the disease is so terrible as to mean that she does not have a minimally acceptable quality of life. In such a case, Parfit argues that the fact that the child could not have existed without that condition does *not* prevent us from concluding that s/he has been harmed. A threshold has been passed in the latter case, which was not breached in the case of the child with congenital syphilis. Many people have taken issue with Parfit’s reasoning. Some object to the idea that conceiving a child whose life is only just worth living is not per se harmful to that child [14]. Others may simply not share Parfit’s person-affecting requirement, in which case the ‘non-identity problem’ is no problem at all. However, my approach is not based on either of these broad objections to Parfit’s arguments. Rather, I am sceptical about the logical connection between the assertion that some lives are not worth living, and the claim that such people are harmed by being conceived.

### Quantifying harm: the sting scale

If we are to make use of the harm threshold in legislation, we must *operationalise* it, and this requires further analysis of where exactly it should be located. Here, there is a difficulty, since Parfit himself resolutely avoids establishing exactly where the threshold might lie. He asserts that one person may be better or worse off than another, but acknowledges, ‘I do not assume that these comparisons could be, even in principle, precise. I assume there is only rough or partial comparability’ [13, p. 357]. Phrases such as ‘worthwhile life’ are used to indicate this rough distinction between those conceptions that are harmful and those that are not in Parfit’s work [13, p. 358]. Yet, the connection between the worthlessness of a life and the fact that a child has been harmed is not demonstrated in Parfit’s analysis. Indeed, based on Parfit’s own comments above, it seems possible that he believed the connection could not be demonstrated.

If, as Parfit suggests, suffering cannot be accurately weighed or gauged, and if there are no precise and objectively accurate means of comparing outcomes, it becomes less obvious that the harm threshold will support a clear mandate for banning *any* uses of reproductive technology. However, it might be argued that we do not need to establish an exact location for the harm threshold—any more than we need to identify the threshold at which night becomes day, or at which a child becomes an adult—all we need do is agree that at some point on a spectrum, these distinctions are manifest. But although this may help in sustaining our intuitive feeling that some reproductive choices are wrong, it does not explain how the harm threshold should be used in practical moral judgments or legislation. In order to operationalise the harm principle, we must at the very least be prepared to adopt a pragmatic approach. Few people may believe it incontrovertibly acceptable to experiment on human embryos up till 14 days, and incontrovertibly unacceptable to do so afterwards. The cut-off point in UK law is pragmatic: since we are not likely to find out exactly where the threshold lies, it must be negotiated and reasons given for setting it here rather than there.

If we want to know whether possibilities such as cloning or the creation of disabled embryos should be forbidden by law, we will at some stage have to ask: *do* these possibilities breach the harm threshold, or *don’t* they? For this reason, it is not enough, in the context of reproductive ethics and legislation, to assume the existence of a harm threshold without considering how it functions and where it is located, even if we accept that these deliberations will be very difficult and may be imprecise. If we could accurately and objectively quantify suffering, we would be better able to identify or at least negotiate where the threshold should be set. By imagining how we would set about locating the harm threshold if this were the case, we can get closer to thinking about how the threshold works. Imagine that suffering can be registered on a scale in units equivalent to wasp stings. A large number of stings might make life intolerable^2^ and would eventually be fatal [15]. Assuming for the sake of argument that 1000 is invariably fatal, somewhere on this scale is the threshold—a point at which life becomes intolerable to the sufferer.

If the threshold is set at, say, 800, we may claim that to conceive a child whose suffering equals or exceeds 800 on the sting scale, *harms* that child. Suppose now that three children, A, B, and C, are conceived by means of experimental reproductive technologies. Each suffers from genetic anomalies resulting from the circumstances of their conception. A’s suffering registers 800 on the scale; B’s measures 799, C’s registers only 1. For Parfit, A has been harmed by being conceived, B has not (and may indeed have been benefited) [16]. Nevertheless, both children suffer, and to a very similar degree. There is only the difference of a single wasp sting between them. Now, comparing B and C, there is an enormous difference between these children’s *suffering*. But the difference in degree in this instance, despite being much greater, is negligible in terms of *harm*.^3^ Neither of these children has been harmed by their conception.

We usually understand the relationship between suffering and harm as being more or less co-linear: whenever suffering increases, so does harm. On this view, the further along the sting scale one is, the more harm one experiences. But this is not what Parfit would have us do. Rather, the harm involved in reproductive choices is non-existent as the suffering of the prospective child increases, until the child reaches the designated point on the scale, and only then harm suddenly materialises. If we accept Parfit’s argument, child B has not been harmed. This is *not* because B does not suffer—Parfit would concede that he suffers a great deal—but *because there is no causal mechanism by which we can understand him to have been harmed*. There is something peculiar going on here in Parfit’s argument: his reason for arguing that most people are not harmed by being conceived is not primarily because they are not suffering *enough*. Rather, it is a metaphysical and logical point: *they could not otherwise have existed.* Yet, Parfit gives us no logical or metaphysical reasons for differentiating between child A and child B. All he gives us is a difference (possibly a very small difference) of degree. There is a far greater difference of degree between the suffering of child B and child C, but for Parfit, we are not entitled to draw any harm-based conclusions from this.

When we uncouple suffering (or pain or sickness) from harm in order to fit in with Parfit’s arguments, it seems to raise questions about what harm *is* if it is not directly connected with pain or suffering in these smaller quantities. It seems that Parfit is using the concept of harm in a rather peculiar way. It is divorced from its normal relationship with suffering, and becomes a mercurial entity, flashing into existence in certain very specific circumstances.

David Heyd argues that ‘non-identity is not a matter of the degree of harm or pain but a conceptual constraint regarding the conditions for making any moral judgment’ [17]. But while Heyd may be right that this is the correct way to think about non-identity, it is undeniable that Parfit moves from making the *logical* claim that existence cannot harm someone in most cases, to the assertion that it *is*, after all, a question of degree. Perhaps Parfit’s point is that suffering, pain, or sickness *can* *cause* harm, though they do not invariably do so. If we take this approach, we might conclude that suffering, pain, and sickness are not bad in themselves, but only insofar as they actually cause harm—and this only happens at a certain point on the scale.

Accordingly, we could separate sickness from harm, and say that although child B, who registers 799 on the scale, is suffering, she is not suffering *harm* per se, because this degree of sickness is not sufficient for her to cross the harm threshold. Yet, this seems very odd. Usually sickness and suffering are regarded as bad in their own right, not solely on the basis that they may be harmful in some additional way. When thinking about existing people, we tend to assume that anything that moves them even a single point up the scale *harms* them. So why is it that there seem to be separate methods of calculating harm, one for existing people, and one for future people? And why are they so different?

Perhaps there is a difference because Parfit and those who draw on his reasoning *offset* the harms of conception against its benefits, whether explicitly or implicitly. I cited Macklin, above, who stated that the harms involved in cloning would have to be very severe indeed in order to ‘outweigh the benefits of life itself’ [2, p. 213]. Macklin and many others assume that to exist is, all other things being equal, a benefit.^4^ The harm threshold is set high in cases of conception because at some level, it is assumed that if conception can harm someone, it can also benefit them. And if this is the case, only where suffering is extremely severe will we deem conception to have been harmful. This may also clarify the fact that the relationship between suffering and harm seems to operate differently among living people. In their case, we cannot offset suffering against the putative benefits of existence: their existence is a given, and any suffering can only reduce their quality of life.^5^ Suffering among the living *is* therefore co-linear with harm.

### Existence as a predicate

If the harm threshold could be identified simply by subtracting suffering from the benefit of coming into existence, this might resolve some of the difficulties encountered earlier. However, the idea that entities can be benefited *or* harmed by being conceived raises another set of problems. These questions resonate with a much older philosophical dispute about what can be encompassed by existence. One of the classical proofs for the existence of God is the ‘ontological argument’, put forward in the 11th century by St. Anselm (see, e.g., [18]), and subsequently in various guises by later philosophers (see, e.g., [19]). Anselm argued that it is possible to have an idea of a being ‘than which none greater exists’. However, he asserted that it is contradictory to suppose that God could exist solely in the understanding, because a greater being *can* be conceived: one which exists in reality as well as in the mind. For Anselm, existence and greatness were encompassed in the verb ‘is’. Since God was great, He must therefore exist, and existing greatness is greater than non-existing greatness. If we conflate existence with other properties as Anselm does, we might conclude that the real child A and child B are slightly different from their imaginary counterparts. When we compare the properties of the imaginary children with the real ones, we add something extra to the real ones over and above the fact of their existence: the property of having been *harmed or benefited* by existing. However, there are difficulties associated with Anselm’s argument, and to the extent that the harm threshold rests on a similar conflation of existence with other properties, the same problems may apply.

Many philosophers and theologians have attacked Anselm’s argument, but the most compelling—and famous—refutation of this argument is probably Kant’s. Kant dismissed the ontological argument on the basis that it erroneously used the word ‘is’ to imply more than simply existence [20, p. B628]. ‘“Being” is obviously not a real predicate; that is, it is not a concept of something which could be added to the concept of a thing. It is merely the positing of a thing, or of certain determinations, as existing in themselves. Logically, it is merely the copula of a judgment. The proposition, “God is omnipotent” contains two concepts, each of which has its object-God and omnipotence. The small word “is” adds no new predicate, but only serves to posit the predicate in its relation to the subject’. Kant illustrates his point with the well-known phrase: ‘a hundred real thalers do not contain the least coin more than a hundred possible thalers’ [20, pp. B626–627]. Yet, this may not be entirely satisfactory. A hundred real thalers as Kant acknowledges, would clearly have more spending power than 100 imaginary ones. Likewise, 100 real wasp stings are much more unpleasant than 100 imaginary ones. Anyone would prefer to have the real thalers, and to forego the real wasp stings if given the option. A little more consideration is required here to do justice to Kant’s analogy.

When we ask *how much* *worse* 100 real wasp stings are than 100 imaginary ones, the answer is, of course, 100 times worse. But to say this means, in fact, nothing more than reiterating that the real stings cause *real* pain, while the imaginary ones do not. The point can be made still more clearly if one asks whether 100 real wasp stings are better or worse than 500 imaginary ones. The pain of the real 100 stings will still hurt more than the imaginary 500. There is no point at which the number of imaginary stings, or thalers would outstrip the real ones. It is for this reason that for Kant, it makes no sense to take the fact that one can buy more with 100 real thalers to indicate that the real money has any property in addition to the conceptual money—apart from its being real.

This, Kant argues, is because existence cannot function as a predicate in the same way that predicates such as ‘great’ or ‘perfect’ do. Between a conceptual object and an actual object that share every property bar existence, there can be no differences apart from existence itself. Nor can existence change the *degree* to which a property is held. The real God is not a few degrees more perfect than a conceptual one. A real strawberry is not a little sweeter than a conceptual one, or a real wasp sting a touch more painful than a conceptual one. If we apply this reasoning to the idea of pain, suffering, and harm, we can see that on Kant’s view, it would be nonsensical to suppose that a real clone’s suffering would register a little higher on the sting scale than an imaginary one that is identical in every other respect. The actual clone’s suffering is real, and the conceptual one’s is not. But their place on the scale is identical.

However, Parfit is not arguing precisely that a real child’s suffering is a notch or two further along on the sting scale when compared to a conceptual one. To establish whether Parfit’s argument falls within the scope of Kant’s refutation, a further two steps are required. The first is to ascertain whether Kant’s points about existence apply to other aspects of existence, such as creation. The second step is to establish whether harm can be construed as a property in such a way as to bring it within the scope of Kant’s argument. I will consider creation first. The act of creation can be construed uncontroversially as the act of bringing something into existence. If this is the case, then whatever is logically true of existence in general should be true of creation insofar as it is the conferring of existence. In other words, if existence itself cannot entail any other property, then it follows that the mere act of giving existence cannot encompass the act of conferring any property—other than existence—on the entity which is created.

Now to address the question of whether ‘harm’ is the kind of predicate that Kant had in mind. Certainly, the word ‘harm’ does not usually function as a predicate in the way that the words ‘great’, ‘round’, or ‘red’ do. It might be thought that because of this, harm cannot be regarded as the kind of predicate that Kant discusses in his argument. Nevertheless, if a child can be harmed by being conceived, as Parfit contends, this harm must logically be connected with some of the properties pertaining to the child. In the case of a cloned child or one with a devastating disability, these properties might include certain genetic anomalies. Thus, although harm itself is not a property, or a predicate in the usual sense, the fact of having a particular genetic makeup *is* a property. The descriptive terms that are often used to indicate genetic anomalies (e.g., Down’s Syndrome or XXY) do function as predicates, in that they qualify an entity. That is, they are logically similar to other predicates such as red or round—they provide additional information about what *kind* of entity is being discussed. The ability to say a child has been harmed is dependent on its having these properties.

If we accept that Kant’s claims regarding existence also apply to *conferring existence*, and that harm, though not in itself a predicate, depends on properties that *are* predicates, we can begin to see the problems inherent in Parfit’s logic from the Kantian perspective. As existence itself cannot entail any other property, similarly the act of conferring existence cannot be identical with the act of conferring any other property. Creating something, or bringing it into existence, is not therefore the same as harming it. If we cannot make X greater, more perfect or more valuable by bringing it into existence, neither can we harm X by bringing it into existence however greatly X may suffer.

### Dealing with Kant’s critics

Those who advocate a harm based approach frequently claim a kind of epistemological superiority over Humean, virtue, or deontological perspectives; yet, seemingly after all, they themselves are mired in a logical fallacy. However, Kant’s refutation of the ontological argument has met with criticism and it is worth considering whether his critics’ comments have a bearing on my application of his reasoning to the harm threshold. Kant appears to regard his rebuttal as conclusive. But for those who are not already sceptical about the ontological argument, Kant’s refutation may be less compelling. Here, I consider objections advanced by two key commentators: Norman Malcolm and Alvin Plantinga.

Norman Malcolm identifies two separate forms of Anselm’s argument, and suggests that Kant’s refutation is only partially effective. The first form of Anselm’s argument deals with ‘existence as a perfection’. For Malcolm, this is fallacious as Kant shows, because in taking existence to be a perfection, it is treated as a predicate. However, Malcolm suggests that Anselm’s second formulation should be understood to claim that a being whose non-existence is *logically impossible* is greater than one whose non-existence *is* possible. This, Malcolm argues, is not dependent on the fallacious assumption that existence is a predicate. Thus, Kant’s refutation only partially demolishes the ontological argument [21].

Malcolm’s point seems reasonable in that the second claim does not raise the same logical problems as the first form of the argument. Something which exists by logical necessity may well be qualitatively different (i.e., has different predicates) from something which does not. However, it appears that only the first formulation of Anselm’s argument, in Malcolm’s analysis is relevant to the Parfitian questions that I am considering here. In Parfit’s case, we are not talking about what might follow from the logical necessity of existence, but about the attributes that existence may entail. Thus, even if Malcolm’s criticism holds, it does not apply to the first category of ontological claim as made by Anselm, and we may be justified in concluding that insofar as Parfit’s claims relate to the first rather than the second formulation, they are effectively dismissed by Kant’s argument.

Alvin Plantinga, however, takes a different line, and his objection is more sweeping than Malcolm’s. Plantinga points out that concepts lack some of the predicates that real objects have. A conceptual horse would not necessarily be a specific height. But an actual horse must be some height. Hence, it would seem that existence *can* entail additional properties, and indeed *must* do so. I suggest that there are two aspects of Plantinga’s critique that are problematic. Firstly, his interpretation of Kant is open to question, and I will put forward an alternative interpretation. Secondly, if one pushes Plantinga’s horse analogy further, it leads to bizarre and implausible conclusions, as I will show.

It is Kant’s choice of money as an example that opens the way for Plantinga’s objection. We rarely think of coins in terms of their individual features. One pound is identical in most respects to every other single pound. Because of this, Plantinga takes it that Kant is describing a sort of conceptual prototype. The realised object, as Plantinga shows with his horse example, will always have some specific properties that a prototype does not. However, there seems to be a difference between Plantinga’s interpretation of a concept and Kant’s—and of the relationship between the concept and its real counterpart. A hundred real thalers coined at a particular mint will bear certain dates and marks; they will have properties independent of their monetary value. For Plantinga, this is the kind of distinction that marks out the conceptual from the real, and gives the lie to Kant’s claim that existence entails no additional properties. Yet, Kant is of course not committed to denying the possibility that a conceptual 100 thalers might also carry certain individual properties in addition to their conceptual worth in financial terms.

The question here is whether Kant is referring to the concept of 100 thalers, as Plantinga suggests, or referring to *this particular 100 thalers,* with all their individual attributes. Kant explicitly stipulates that even if we could identify and enumerate *every* property pertaining to the imaginary object, existence still would not function as a predicate in the way that others do: ‘By whatever and by however many predicates we may think a thing—even if we completely determine it—we do not make the least addition to the thing when we further declare that this thing *is’* [20, p. B628]. Plantinga denies that this is the case. He discusses a set of objects, which are pink—a predicate. Of this set, some of those objects exist, while others are imaginary. This example seems to swap the functions of existence and pinkness, in that while it adds nothing to the concepts to say that they are pink (they are pink by necessity) it *does* add something to say whether or not they exist, since some—like Valhalla, which has pink walls—are imaginary, while others are not. But this is trivial, Plantinga argues, as it boils down to the statement that ‘*all existent members of D exist* is necessarily true, but *all existent members of D are pink* is not’ [22].

There is not scope in this paper to fully explore the nuances of Plantinga’s reasoning, and not every aspect of his argument is relevant for my purpose. However, it is clear that for Plantinga the relationship between the identity of the objects and the necessity of their predicates is crucial. It is this that is problematic in the non-identity problem: the predicate (harm) is necessarily associated with the child’s identity, and has become entwined with existence in such a way that we find it hard to separate them. What I want to do here is to show that if Plantinga’s rejection of Kant’s refutation holds, that is, if we *can* associate existence with other properties, and thus also associate creation with conferring other properties, this leads to further logical difficulties.

If we accept that existence can function as a predicate, as Plantinga suggests, and that existence confers additional properties, we can ask which properties are being conferred along with existence when we bring something into being. Suppose there were a button one could press to bring things or people into existence. As Plantinga notes, we cannot bring a horse into existence that lacks the property of height altogether; therefore, in pressing the button to create a horse let us agree that we also confer on it the property of being, say, 15 hands high. Now suppose that some prospective parents are hoping to create a child with a devastating disability. They might also wish for their child to have red hair and to be a girl. For the sake of argument we will assume this disability is so terrible that the child’s suffering would equal or exceed 800 on the harm scale. Parfit’s argument is that to create such a child would be to harm it. The parents envisage the child, press the button, and a child springs into being. The child is disabled and red haired and female.

The parents have conferred existence on this child. On Parfit’s view—which Plantinga’s argument would seem to support—they have also harmed it. I turn now to the redness of the hair. If we can say the parents have disabled the child, and thereby harmed it, by bringing it into existence, it is no logical leap to say that they have also reddened its hair. Indeed, we might also say they have feminised it, shortened its nose, enlarged its ears, fattened its stomach, and broadened its forehead. This makes the idea of creation redundant: seemingly, it can be replaced with a list of other actions, e.g., reddening, shortening, etc., until every property pertaining to the entity in question is accounted for. On this view, one does not create a child with the properties of being disabled, red-haired, and female. Rather, one disables (or harms), reddens, and feminises an entity.

However, some might object by saying that this is not what Parfit argues: he specifically tells us the property must exceed a certain threshold for us to regard the act of creation as being synonymous with conferring the property. Similar approaches could be applied to other scaleable attributes. On this view, it might seem that only if registering above a certain point on a scale, has a child’s hair been reddened, or its nose shortened and its ears enlarged in its creation. This means that only in certain circumstances is the act of creation accompanied by the reddening of a redhead or the fattening of a fat person (provided they meet the required threshold). If we want to be accurate in determining exactly what we can be said to be doing when we bring something into being, we may be faced with the task not only of identifying the harm threshold, but many other thresholds too. Of course, Parfit does not explicitly say this, but it is not clear how he could avoid it, since his claim is that the possibility of affecting something through its creation is somehow related to the degree to which it holds that eventual property.

Turning back to Plantinga’s example, suppose we decide to create a small horse: can we say that we have *ponified* the creature by creating a small one rather than a large one? If we accept the threshold view, the answer will depend on the animal’s eventual height. If it is only 13 hands high, we have indeed ponified it, since what would have been a horse, if it had been larger, is only a pony at 13 hands. This is a threshold of the same sort as Parfit’s harm threshold. Applying Parfit’s reasoning to Plantinga’s example, we could conclude that not only have we created a pony but we have also *ponified* it, and that when we create a disabled child, we have *disabled* it. But in both cases, when we ask *what* is the entity that we have ponified or disabled, the flaw in this reasoning becomes apparent. We have ponified a pony; we have disabled a disabled person. But this will not work: we cannot perform an act on an entity that makes them what they already and necessarily are.

In all, Plantinga’s objection to Kant does not seem to circumvent the logical problems associated with conflating existence with other properties. We end with a circular statement of exactly the sort that Plantinga objects to in his analysis of Kant [22]. Thus far, then, it looks as though the claim that beyond a certain threshold we harm children by conceiving them is indeed based on a fallacy. However, in reality of course, children are not conceived mentally and then brought into being at the touch of a button. The processes involved are more complex and take longer. Kant’s arguments may therefore be more difficult to sustain in the messier real-world context of bringing children into existence. If one really did want to create a child with a devastating disability, one could specially select a particular egg and sperm carrying a genetic mutation that would inevitably cause that condition (which, for the purposes of this argument, guarantees a greater than 800 score on the scale). The sperm could then be injected into the egg, and the embryo implanted into a woman’s uterus. We then have to establish whether any of these acts constitute disabling and, thus, harming the child.

Deciding to conceive a disabled child, even if it is regarded as being morally reprehensible, cannot be construed as disabling a child. The selection of a particular egg and sperm likewise is not a process that can be regarded as harming a child. The act of fertilisation is clearly key to the *creation* of the child—it cannot exist without this act—but again, to claim that this act of fertilisation is the same as an act of harming is simply not convincing. We cannot identify the entity that we are supposed to be acting upon—an egg is not harmed by being fertilised. At no part of the process is there any act that—if it had been omitted—would have meant that this particular child was free of disability. The potential child that could be formed from that egg and sperm could not exist and not be disabled.

Extrapolating from the examples given above to the more abstract question of harm, it is equally implausible to view any of these stages as *harming* a child, any more than it constitutes *shortening* or *fattening* a child or *ponifying* a horse. The reason I have used the specific example of ‘disabling’ in my argument up till now is largely because the rather abstract nature of the word ‘harm’ lends itself to logical vagueness, which can more easily be demonstrated by substituting a specific harm such as disabling. In case anyone should object to this manoeuvre, it can easily be defended by observing that in my hypothetical example of the disabled child, there is no suggestion that any other harm or suffering is involved aside from the disability itself. Therefore, if the child is supposed to be harmed solely by virtue of being disabled, *the act of harming must be the act of disabling*. But since, as I have shown, no act of disabling has been carried out, neither has any act of harming.

## Conclusion

In this paper, I have asked whether there is a harm threshold that can be used in determining which reproductive choices and techniques should be allowed and which should be illegal. According to my argument, it is illogical to equate existence with either harm or benefit. Consequently, there is no specific act that can be construed as harming a future child when the child’s condition is directly linked with the circumstances surrounding its conception. Nor can these questions be a matter of degree, since the logical and metaphysical constraints that prevent us from concluding that a child born with a moderate amount of suffering has been harmed apply equally to all cases of creation. For these reasons, then, I would suggest that novel reproductive technologies cannot be condemned on the grounds that children conceived through these means are harmed. *There is no harm threshold.*

This is not to say that it is impossible to perform any acts now that will harm people who are not yet conceived. Parfit addresses this in his discussion of harm to future generations. If one leaves a piece of jagged glass in a wood, and a child born some years later hurts himself on it, one’s action has caused this harm [13, p. 356]. But this cannot serve as a parallel to causing harm to a future person through the very activities that bring about his existence. Note that in Parfit’s example, if one chooses *not* to leave the glass in the wood, this choice circumvents an injury to the child. The child thus benefits from the omission. In contrast, if we choose *not* to inject the sperm into the egg, we do not thereby prevent this child from being disabled. In fact, there is no action that can be taken to circumvent disability in this particular child. Thus, unlike the child in the woods in Parfit’s example, this child cannot be harmed or benefitted by the omission of the acts in question.

As I have suggested, much of the appeal of harm-related legislation seems to lie in the idea that it is less subjective and more empirically sound than other legal approaches to reproductive technologies. But Parfit’s suggestion that there are no precise or objective measurements to prove his conjectures suggests that we are simply obliged to take his word for it that this is how things are. Parfit argues that one state can be compared with another and—broadly—be deemed better or worse. But since he provides no measures, and indeed, argues that *there can be no definitive proof*, it is not clear how these ideas can play a part in harm-based legislation. Moreover, the very concept of the harm threshold—on which his argument rests—is logically untenable.

The concept of the harm threshold has been inadequately operationalised by those who espouse it. This is largely due to a failure to identify the flaws in the arguments concerned and to recognise the fallacies involved. Many of these difficulties arise from the demand for an empirically sound way of evaluating a life and legislating for the morality of reproductive decision making. In an area of such complexity, if we could rely on a relatively simple concept such as the harm threshold to help us determine which reproductive choices are morally or legally acceptable, it would make things easier. However, as Bernard Williams has pointed out, this is the perennial problem of a utilitarian approach. Those things which are not easily quantifiable must either be hacked into some form that enables them to be fitted into the theory or be omitted altogether [23, p. 103]. One of the attractions of the Millian harm principle in this context is its apparent avoidance of all but the most basic moral or value judgments: those connected with health or clinical outcomes for the child. Yet, if it is impossible to demonstrate that children are harmed by being born, this powerful tool for justifying legislative and regulatory restrictions is in fact of no use at all.

This may seem to be an unsatisfactory conclusion. It seems to leave us wide open for all and any reproductive choices that people might seek to pursue with the help of reproductive technologies. However, there are a number of other possibilities that could be considered. In my discussion above, I noted that deciding to conceive a disabled child is not the same as disabling a child. Nevertheless, many people believe that making such a decision is morally wrong. The challenge for those who do believe so is to establish whether there are grounds for this belief other than that the child has been harmed. The lure of Mill’s harm principle is so strong that it can be tempting to assume that any moral wrongdoing must be explicable in terms of harm caused to another human being. Yet, of course, even Mill himself did not hold this to be the case. It is simply that Mill believed that preventing harm to others is one of the only possible justifications for restricting people’s freedom.

The use of new reproductive technologies has an impact on people other than the children who are born through this technique. Such technologies could cause broader harm—perhaps to society itself. Here, the question of disability or disease comes in again, not in the context of whether the child has been harmed through its conception, but in the context of resources expended on sustaining and treating children with extensive healthcare needs. For those who are wedded to a person-affecting morality, there may be further work to be done to show how a broader harm-based approach could be applied to reproductive legislation. Though the harm threshold proves illusory, we do not have to jettison the harm principle altogether. However, the claims that can be made for this principle in the context of reproduction are very much more modest than many of its proponents assume.

## Footnotes

1 Some would argue that reproductive cloning is *not* intuitively immoral. Here, I do not seek to demonstrate that it is, but merely use this as one example of a procedure whose results are unknown and likely to be risky.

2 There are questions here about the duration of the suffering and its association with harm. I cannot go into this fully in this paper, but I acknowledge that it is a complex issue. For the sake of my wasp sting harm scale, I am assuming that the suffering caused is persistent throughout one’s life.

3 Parfit, of course, sets out circumstances in which he argues that the choice of the short sighted child is a morally preferable choice to that of the cystic fibrosis child. But this difference is not based on the idea that either child is harmed by coming into existence; rather it relies on the idea that the parents had a choice as to which child to conceive, and should have chosen the one who will suffer less. There is much to be said about Parfit’s same-number choice analysis, which raises its own problems of scope. However, for the purposes of this paper, I will focus on the idea that parents are *not* choosing between different options. They will reproduce by cloning or not at all. Thus, I am not comparing the decision of parents to have a child with CF or short sightedness, but simply whether, in each case, the decision to have the child with that condition can be said to have harmed it.

4 The relationship between harm and benefit in Parfit’s argument is complex. Parfit stipulates that he thinks it plausible that bringing someone into existence *can* benefit them—and also to argue that it *cannot*. Despite his stated ambivalence on this point, the thrust of his argument suggests that he tends towards the view that existence *can* benefit [13].

5 Of course, there may be arguments to suggest that suffering does not invariably reduce one’s quality of life, and even that it may enhance it. However, even if this point is accepted, a deliberate act of increasing an individual’s suffering is generally perceived to be wrong.
